# Changes in peak expiratory flow rates using two head-tilt/chin-lift maneuver angles in young healthy conscious volunteers

**DOI:** 10.1371/journal.pone.0224155

**Published:** 2019-10-18

**Authors:** Sion Jo, Jae Baek Lee, Youngho Jin, Taeoh Jeong, Jaechol Yoon, Boyoung Park, Jin Mu Jung

**Affiliations:** 1 Department of Emergency Medicine, College of Medicine, Chonbuk National University, Jeonju, Jeollabuk-do, Republic of Korea; 2 Research Institute of Clinical Medicine of Chonbuk National University, Jeonju, Jeollabuk-do, Republic of Korea; 3 Biomedical Research Institute of Chonbuk National University Hospital, Jeonju, Jeollabuk-do, Republic of Korea; 4 Department of Medicine, Hanyang University College of Medicine, Seoul, Republic of Korea; 5 Division of Mechanical Design Engineering, College of Engineering, Chonbuk National University, Jeonju, Jeollabuk-do, Republic of Korea; Weill Cornell Medical College, UNITED STATES

## Abstract

**Background:**

The head-tilt/chin-lift (HT/CL) maneuver is simple and routinely used to open a closed upper airway.

**Objectives:**

It has yet to be determined whether increasing the HT/CL angle further would be beneficial.

**Methods:**

We enrolled 60 (30 males) 20-year-old conscious participants. Pre-HT/CL, post-HT/CL #1, and post-HT/CL #2 positions were defined as positions in which the angle between the ear–eye line and the horizontal line was 80°, 65°, and 50°, respectively. Peak exploratory flow rates (PEFRs) pre-HT/CL, post-HT/CL #1, and post-HT/CL #2 positions were recorded continuously at 1-minute intervals (one set). Five sets of measurements were performed (total, 15 measurements for each participant).

**Results:**

We analysed 900 measurements (180 sets). The mean PEFRs pre-HT/CL, post-HT/CL #1, and post-HT/CL #2 positions were 348.4 ± 96.9, 366.4 ± 104.9, and 378.8 ± 111.2 L/min (percentage change compared to pre-HT/CL, 5.2% and 8.7%), respectively. Significant differences were observed among pre-HT/CL, post-HT/CL #1, and post-HT/CL #2 positions in all participants, as well as in subgroup classified according to sex, and medians of height, body weight, and body mass index.

**Conclusion:**

Our findings suggest that a greater HT/CL angle would be beneficial, as the PEFR increased gradually. The decreasing manner in the PEFR increase with the HT/CL angle implies the existence of an angle threshold beyond which there were no further benefits in airflow, indicating a minimum in airway resistance. A HT/CL maneuver may be appropriate until locking the atlanto-occipital and cervical spine joints in extension occurs and the chest (sternal notch) begins to rise.

## Introduction

The head-tilt/chin-lift (HT/CL) maneuver has long been recommended as a method of opening a closed airway in unconscious patients without head or neck trauma. Many international guidelines have supported the use of this maneuver; however, there is limited clinical data available in relation to the maneuver [1–4]. While the HT/CL maneuver is very simple in practice, questions remain concerning the HT/CL angle because the HT/CL maneuver is not like turning a switch on and off. First, the angle in which to open a closed airway remains to be determined. Second, after a closed airway has opened, it has not been established whether additional angulation would be beneficial (angulation dependency of an opened airway). These questions have yet to be resolved because unconscious patients require immediate emergency management and are not able to be measured or examined for purposes other than those required during such emergencies.

However, whether additional angulation would be beneficial may possibly be evaluated among participants whose upper airway is already open and who are conscious, as this can be directly determined through measuring the airway flow rate via the upper airway. Under the same exploratory power conditions, resistance and flow rates are inversely proportional. If airway resistance decreased, the airway flow rate would be faster, and if airway resistance increased, the airway flow rate would be slower. Based on this rationale, we previously examined the peak exploratory flow rate (PEFR) among conscious participants pre- and post-HT/CL. The PEFR increased 9.6% with a 15° HT/CL maneuver angle [5]. The angle between the ear-eye line (EEL) and the horizontal line (HL) (the EEL and HL angle) was 80° pre-HT/CL and 65° post-HT/CL in that study.

We hypothesized that there would be an angle-dependent increase in post HT/CL PEFR in conscious participants. In this study, we selected angles between the EEL and the HL of 80°, 65°, and 50° to examine this hypothesis.

## Materials and methods

### Ethical considerations

**Fig 1:**
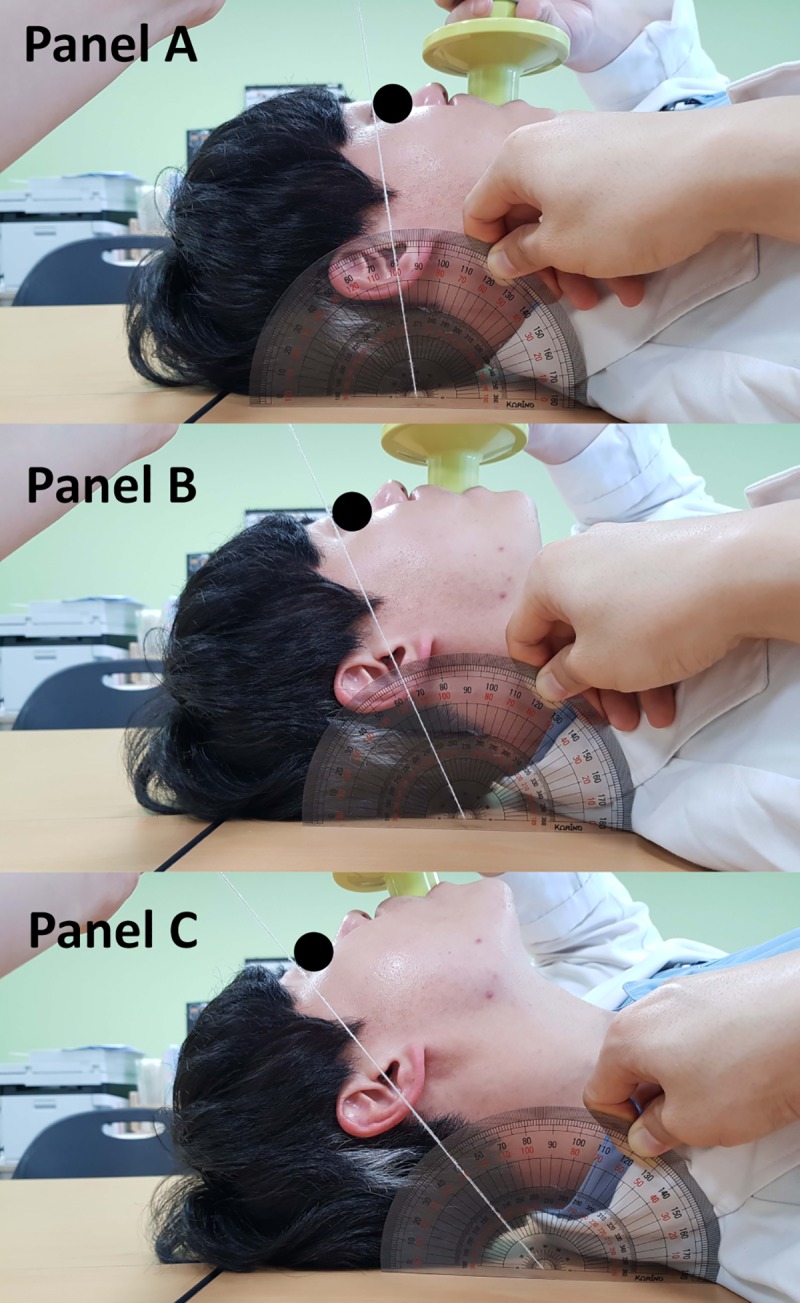
**Pre-HT/CL position (panel A, EEL and HL 80°), post-HT/CL #1 position (panel B, EEL and HL 65°), and post-HT/CL #2 position (panel C, EEL and HL 50°).** The displayed model is not a study participant. Abbreviations: EEL, ear-eye line; HL, horizontal line; HT/CL, head-tilt/chin-lift maneuver.

This study was approved by the Institutional Review Board of a Chonbuk National University Hospital. After each participant was provided with a thorough explanation of the study procedures using a visual supplement, participants voluntarily provided written informed consent for enrollment. The individual pictured in Fig 1 has provided written informed consent (as outlined in PLOS consent form) to publish their image alongside the manuscript.

### Participants selection

Our previous study measured the PEFRs pre- and post-HT/CL. The mean pre-HT/CL PEFR was 316.1 ± 87.6 L/min, and the mean post-HT/CL PEFR was 346.5 ± 94.7 L/min [5]. Based on these results, we required a sample size of 60 participants to identify a mean difference of 30 L/min with 80% power and an alpha error of 5% for a no drop-out rate. As the PEFR reference value differed according to sex, we included 30 male and 30 female participants.

Between 1 November and 31 December 2018, 60 healthy 20-year-old participants were enrolled. Exclusion criteria were as follows: 1) medical or nursing students who might possibly have had a conflict of interest related to the authors’ affiliation with the School of Medicine; 2) participants with concurrent upper or lower respiratory infections such as pharyngitis, bronchitis, or pneumonia; 3) participants with chronic airway diseases such as asthma or chronic obstructive pulmonary disease; 4) participants with pulmonary tuberculosis or related complications; 5) participants who were obese (body mass index [BMI] of ≥25) or underweight (BMI <18.5); 6) participants with comorbidities such as hypertension, diabetes mellitus, chronic liver disease, chronic renal disease, cardiovascular disease, cerebrovascular disease, or malignancy; 7) participants with known sleep apnea with a possibly distorted upper respiratory airway; 8) participants with a condition limiting the adoption of a supine position such as scoliosis; 9) participants with a condition limiting the adoption of the HT/CL position, and; 10) participants with other conditions deemed as inappropriate for study participation by the authors [5].

### Procedures and measurements

The PEFR represents a person’s maximum speed of expiration, measured using a peak flow-meter. In this study, the PEFR was measured using a commercial hand-held device specifically designed to measure the PEFR (MicroPeakTM, CareFusion, Basingstoke, UK). The measurable range is between 60 and 900 L/min and there is a scale increment of 10 L/min. The PEFR values were rounded to the nearest one-tenth.

The pre-HT/CL position was defined as the position at which the angle between the EEL and HL was 80° (Fig 1). Post-HT/CL #1 and #2 were defined as the position at which the angle between the EEL and HL was 65° and 50°, respectively (Fig 1). The individual pictured in Fig 1 has provided written informed consent (as outlined in PLOS consent form) to publish their image alongside the manuscript.

Data comprising participants’ sex, height, and body weight were collected. Height and body weight were measured at the study site. To reflect the upper airway characteristics of each participant, the lip-mental distance (LMD), the mental-hyoid distance (MHD), and the hyoid-thyroid notch distance (HTD) were measured.

Each participant was provided with a new disposable mouthpiece and was permitted to practice freely, prior to commencement of the study measurements, to familiarize themselves with the PEFR measurements.

### PEFR measurement protocol

PEFR was measured according to the following steps. Steps 2–7 were repeated 5 times to obtain 5 sets of measurements for the supine and HT/CL positions:

1. Each study participant was placed in a supine position on a wooden plinth and was required to remain at rest for 1 minute.
2. The PEFR was measured in the pre-HT/CL position.
3. Each participant was placed in the post-HT/CL #1 position and remained at rest for 1 minute.
4. The PEFR was measured in the post-HT/CL #1 position.
5. Each participant was placed in the post-HT/CL #2 position and remained at rest for 1 minute.
6. The PEFR was measured in the post-HT/CL #2 position.
7. Each participant was placed in the pre-HT/CL position and remained at rest for 1 minute.

Each participant was placed in position by the researcher. Participants were encouraged to breathe out with maximal effort repeatedly when the PEFR was measured.

### Statistical analysis

Discrete data are presented as counts and percentages. Continuous data are presented as the median and interquartile range (IQR), or as the mean and standard deviation if distributed normally.

To compare the PEFR values at pre-HT/CL, post-HT/CL #1, and post-HT/CL #2 for all the participants and in the subgroups classified according to sex, median height, median body weight, and median BMI, we used repeated measured analysis of variance (RM ANOVA).

When sum of a participant’s PEFR at post-HT/CT #1 decreased than that at the pre-HT/CL position, the participant was classified as a non-responder. Also, when sum of a participant’s PEFR at post-HT/CT #2 decreased than that at the post-HT/CL #1 position, the participant was classified as a non-responder. As such, there were three non-responder groups, namely, non-responders to HT/CL #1, non-responders to HT/CL #2, and total non-responders comprising a combination of the aforementioned two non-responder groups. To compare characteristics between responders and non-responders, the student’s t-test was used for normally distributed variables and the Mann–Whitney U-test was used for non-normally distributed variables. For categorical data, the chi-square test or the chi-square test with Fisher’s exact test for 2 × 2 tables was used.

The results were considered significant at a threshold of p < 0.05 (two-tailed). All statistical analyses were conducted using R version 3.4.3 (R Foundation for Statistical Computing, Vienna, Austria) and SAS 9.1 (SAS Institute Inc., Cary, NC, USA)

## Results

### Patient demographics

A total of 900 measurements were collected, comprising 300 pre-HT/CL, 300 post-HT/CL #1, and 300 post-HT/CL #2 measurements. The study participants had a median height of 168.0 cm [IQR 162.0;175.5], and a median body weight of 61.5 kg [52.5;67.5]. The median BMI was 21.3 kg/m2 [19.8;23.0] (Table 1). The median LMD, MHD, and HTD was 4.5 cm [4;4.5], 5.0 cm [4.5;5.3], and 2.0 cm [1.5;2.5], respectively (Table 1).

**Table 1:** Baseline characteristics of the subjects values are presented number with percent or median with interquartile range.

Variables	Total group
Number	60
Male	30 (50)
Height (cm)	168.0 [162.0;175.5]
BW (kg)	61.5 [52.5;67.5)
BMI (kg/m^2^)	21.3 [19.8;23.0]
LMD (cm)	4.5 [4;4.5]
MHD (cm)	5.0 [4.5;5.3]
HTD (cm)	2.0 [1.5;2.5]

Abbreviations: BW, body weight; BMI, body mass index; LMD, lip lower margin-mental distance; MHD, mental-hyoid distance; HTD, hyoid-thyroid notch distance.

### Change in the PEFR post HT/CL

**Fig 2:**
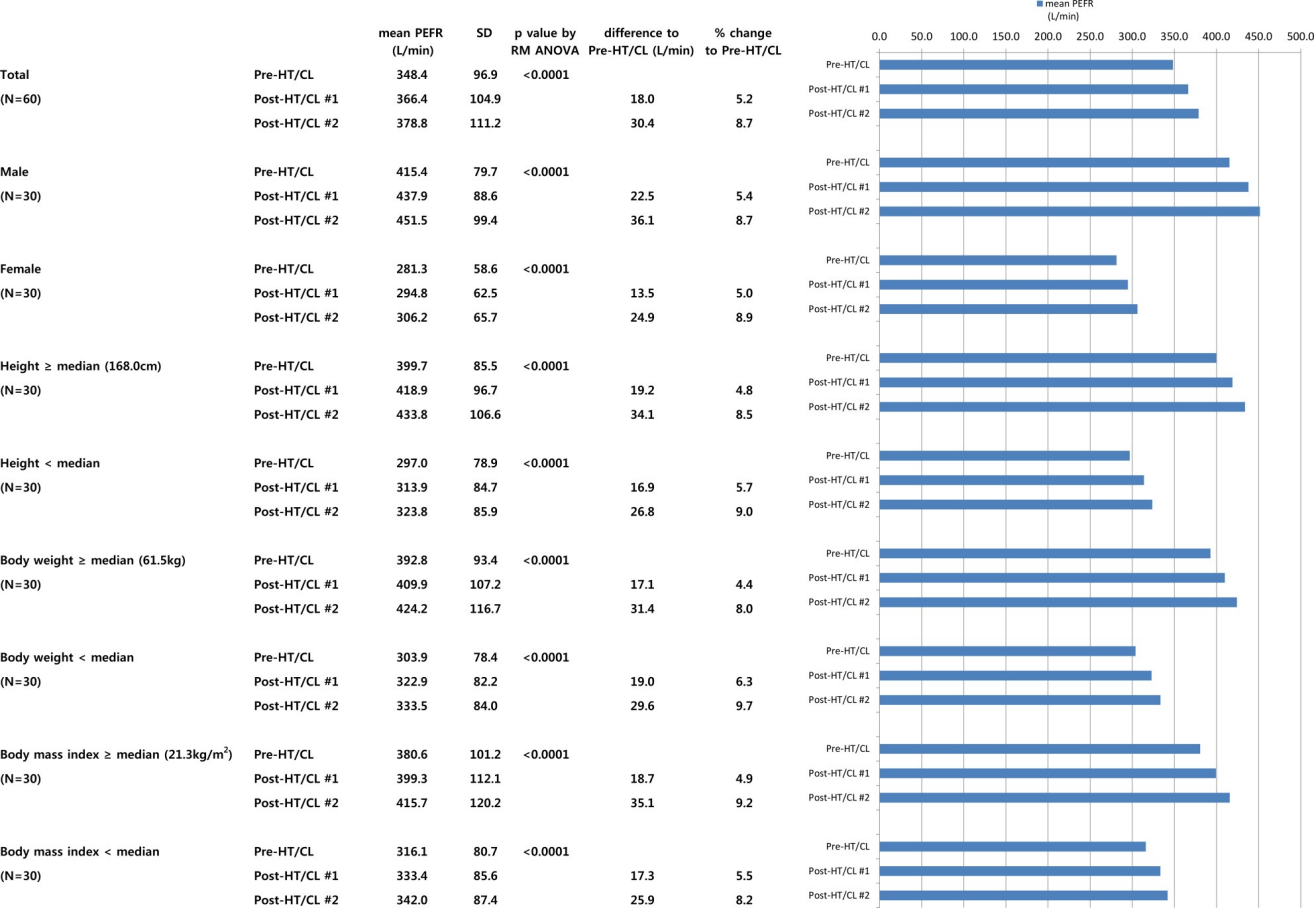
Main result of this study. **A repeated measured analysis of variance was used to compare PEFR values for pre-HT/CL, post-HT/CL #1, and post-HT/CL #2.** Abbreviations: HT/CL, head-tilt/chin-lift maneuver; PEFR, peak exploratory flow rate; RM ANOVA, repeated measured analysis of variance; SD, standard deviation.

Fig 2 shows the main results of the present study. The mean PEFRs pre-HT/CL, post-HT/CL #1, and post-HT/CL #2 were 348.4 ± 96.9, 366.4 ± 104.9, and 378.8 ± 111.2 L/min (percentage change compared to pre-HT/CL, 5.2% and 8.7%), respectively, for all participants. Significant differences were observed among pre-HT/CL, post-HT/CL #1, and post-HT/CL #2 measurements in all the participants (P < 0.0001). The mean PEFR showed a similar gradual increase in the subgroups classified according to sex, and the medians of height, body weight, and BMI, with significant differences in all participants. However, the percentage change post-HT/CL #2 compared to pre-HT/CL levels was less than double the percentage change post-HT/CL #1 compared to pre-HT/CL levels, indicating a decreasing trend in the PEFR increase.

### Non-responders to HT/CL

**Fig 3:**
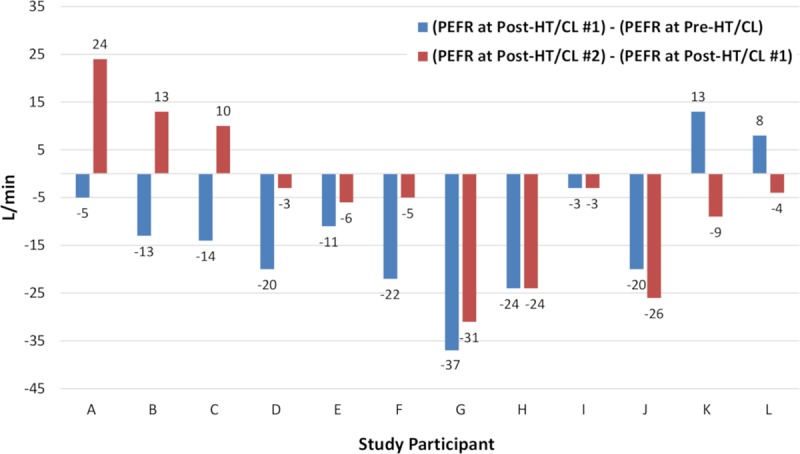
Difference in PEFRs pre-HT/CL, post-HT/CL #1, and post-HT/CL #2 among non-responders. Abbreviations: HT/CL, head-tilt/chin-lift maneuver; PEFR, peak exploratory flow rate.

There were 10 (16.7%) non-responders to HT/CL #1 (Fig 3. Participants A to J). Among those, the PEFR increased at post-HT/CL #2 in 3 participants (Fig 3. Participants A, B, and C) and decreased in 7 participants (Fig 3. Participants D to J). Amount of PEFR decrease became smaller in 4 participants (Fig 3. Participants D to G), remained constant in 2 participants (Fig 3. Participants H and I), and became larger in 1 participants (Fig 3. Participants J). There were 9 (15.0%) non-responders to HT/CL #2 (Fig 3. Participants D to L). Among those, the PEFR change was positive in 2 participants (Fig 3. Participants K and L). Amount of PEFR decrease became smaller in 4 participants (Fig 3. Participants D to G), remained constant in 2 participants (Fig 3. Participants H and I), and became larger in 1 participants (Fig 3. Participants J). There were no significant differences in sex, height, body weight, BMI, LMD, MHD, and HTD between the three responder and non-responder groups, which comprised the non-responders to HT/CL #1 and the responders, the non-responders to HT/CL #2 and the responders, and the total number of non-responders and the responders (Table 2).

**Table 2:** Comparison of baseline characteristics between HT/CL responders and non-responders. Values are presented number with percent or median with interquartile range.

Variables	Responder to HT/CL #1	Non-Responder to HT/CL #1	p-value	Responder to HT/CL #2	Non-Responder to HT/CL #2	p-value	Responder to both HT/CL	Non-responder Total	p-value
Number	50 (83.3)	10 (16.7)	-	51 (85.0)	9 (15.0)	-	48 (80.0)	12 (20.0)	-
Male	26 (52.0)	4 (40.0)	0.729	26 (51.0)	4 (44.4)	1.000	25 (52.1%)	5 (41.7%)	0.747
Height (cm)	170.0 [162.0;175.0]	167.5 [165.0;178.0]	0.558	169.0 [161.0;175.0]	170.0 [167.0;176.0]	0.335	169.5 [161.5;174.5]	169.0 [165.0;177.0]	0.370
Height ≥ median (168.0cm)	26 (52.0)	4 (40.0)	0.729	25 (49.0)	5 (55.6)	1.000	24 (50.0%)	6 (50.0%)	1.000
BW (kg)	58.7 [52.9;70.2]	60.9 [58.3;70.7]	0.226	58.8 [53.4;69.8]	61.7 [58.3;70.7]	0.282	58.7 [52.8;69.8]	60.9 [57.2;70.7]	0.202
BW ≥ median (61.5kg)	24 (48.0)	6 (60.0)	0.729	24 (47.1)	6 (66.7)	0.470	23 (47.9%)	7 (58.3%)	0.747
BMI (kg/m^2^)	21.1 [19.8;22.7]	21.8 [20.9;22.7]	0.204	21.2 [19.9;22.7]	21.3 [20.9;22.7]	0.597	21.1 [19.8;22.7]	21.8 [20.6;22.8]	0.288
BMI ≥ median (21.3kg/m^2^)	24 (48.0)	6 (60.0)	0.729	25 (49.0)	5 (55.6)	1.000	23 (47.9%)	7 (58.3%)	0.747
LMD (cm)	4.5 [4.0;5.0]	4.3 [4.0;4.5]	0.469	4.5 [4.0;5.0]	4.5 [4.0;4.5]	0.658	4.5 [4.0;5.0]	4.3 [4.0;4.5]	0.430
MHD (cm)	5.0 [4.5;5.0]	5.0 [4.5;5.5]	0.318	5.0 [4.5; 5.5]	4.5 [4.5; 5.0]	0.741	5.0 [4.5; 5.2]	4.8 [4.5; 5.2]	0.768
HTD (cm)	2.0 [1.5;2.5]	2.0 [1.5;2.5]	0.617	2.0 [1.5; 2.5]	2.0 [1.5; 2.5]	0.482	2.0 [1.5; 2.5]	2.0 [1.5; 2.5]	0.635

BW, body weight; BMI, body mass index; LMD, lip lower margin-mental distance; MHD, mental-hyoid distance; HTD, hyoid-thyroid notch distance

## Discussion

In the present study, the PEFR gradually increased post-HT/CL #1 and post-HT/CL #2 compared to the pre-HT/CL PEFR, with increases of 5.2% and 8.7%, respectively, which showed that a greater HT/CL angle would be beneficial for airway flow. A notable number of participants showed a decrease in the PEFR post-HT/CL #1; however, the PEFR increased or declining trend slowed down in many non-responders at post-HT/CL #2. There was a decremental trend in the PEFR increase in the HT/CL angle that indicated an angle threshold beyond which there were no further benefits in airflow.

The HT/CL maneuver was first described by Peter Safar almost 70 years ago [6], based on data from 50 spontaneously breathing unconscious patients under anesthetic control [7]. He noted that when the neck was flexed so that the chin touched the chest, the air passage through the throat was completely blocked, and that lifting the chin resolved the airway obstruction in 50% of patients. The remaining 50% of patients required the jaw-thrust maneuver or insertion of an oropharyngeal airway or both [7]. Thereafter, limited data have been reported regarding the HT/CL maneuver, and all studies have derived data involving controlled unconscious patients [8–11]. No study has evaluated the performance of the HT/CL maneuver among uncontrolled (or unexpected) unconscious patients who had been actual recipients of the HT/CL maneuver. This is likely to have been because such patients require immediate emergency treatment and are not readily able to be examined for purposes other than treatment.

Thus, due to a lack of available data and the unfeasibility of further study, basic questions concerning the HT/CL maneuver remain unresolved, particularly concerning the HT/CL angle. First, the HT/CL angle required to open a closed airway remains to be determined. Considering individual anatomical airway variations, the HT/CL angle to open a closed airway is likely to vary and not facilitate the use of a fixed angle. Applying a principle or a formula that corresponds to an individual’s anatomical characteristics may be appropriate. Second, it remains to be determined whether further increasing the angle after a closed airway has been opened would be beneficial. In this case, measuring airway flow rate pre- and post-the HT/CL maneuver would likely be helpful in addressing this matter.

**Fig 4:**
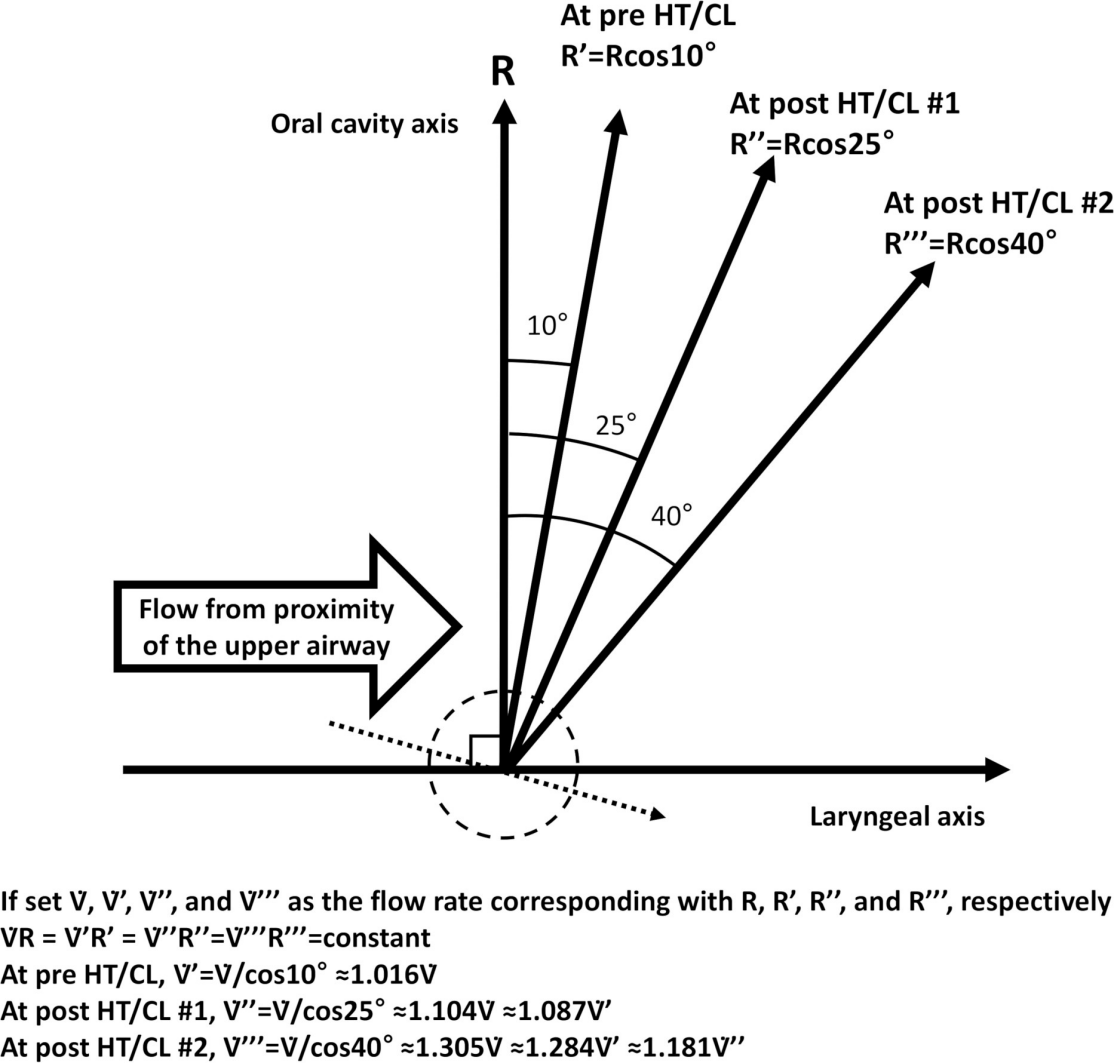
Schematic mathematical model showing the relationship between airway resistance (R) and airway flow rate (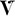) pre-HT/CL, post-HT/CL #1, and post-HT/CL #2. Two points of intersection exist in the dashed round, one point is where the laryngeal axis intersects with the pharyngeal axis, and the other point is where the pharyngeal axis intersects with the oral cavity axis. The dotted arrow indicates deviation of the laryngeal axis according to the rise of the chest. Abbreviations: HT/CL, head-tilt/chin-lift maneuver; R, airway resistance; 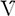, airway flow rate.

The total length of the upper airway remains unchanged pre- and post- the HT/CL maneuver because the HT/CL is an angular movement, not a length movement. The sectional area of the participants’ upper airway was presumed to be constant because they were conscious. If force to the airway is constant and presuming the sectional area of upper airway is constant, pressure to the airway is also constant. Then, if pressure to the airway is constant, resistance and flow rates are inversely proportional (Ohm’s law). Therefore, if the PEFR increased following the HT/CL maneuver, this suggested that airway resistance had decreased. Our schematic mathematical model is shown in Fig 4. The PEFR post-HT/CL #1 was estimated to be 1.087 greater than that of the pre-HT/CL rate. This estimation is supported in the findings of our previous study, which showed a similar percentage change to our estimated change (9.6%).

In the present study, PEFR percentage changes at post-HT/CL #1 and post-HT/CL #2 to pre-HT/CL showed a gradual increase (5.2% and 8.7%, respectively); however, the amount of increase in the PEFR showed decreasing trend. Compared to our previous study findings, we noted the following differences: (1) the percentage of change in the PEFR at post-HT/CL #1 (5.2%) did not reach that of the previous value (9.6%), nor did the estimated value (8.7%), and; (2) a significant gap was found between the percentage change in the measured PEFR (8.7%) and in the estimated PEFR (28.4%) at post-HT/CL #2. Regarding the first difference, we considered the study plinth used to position the participants to be a relevant factor. In the previous study, we used a PVC foam mattress on a wooden plinth for comfort and to relax the participants. However, in this study, the participants lay on a wooden plinth with no padded foam mattress to better reflect an actual collapse scene. In the previous study, the angle between the laryngeal axis and the oral cavity axis would be well matched to that of the schematic mathematical model. However, in this study, the laryngeal axis would be deviated to the right-sided downwards direction (dotted axis in Fig 4) during the HT/CL maneuver because we considered that the chest would be more likely to rise on the hard board than on a soft padded board when the head was tilted. Therefore, the actual acute angle between the laryngeal axis and the oral cavity axis would be greater than the estimated angle, which led to a decrease in the measured PEFR than in the estimated PEFR. We approached the second difference in a similar way. If the laryngeal axis is fixed, a greater HT/CL angle will result in a sharp increase in the PEFR, as shown in the schematic mathematical model. However, the measured PEFR value showed a diminishing increase in the PEFR, which implied the existence of a threshold angle in terms of airflow increase. We considered that the laryngeal axis deviation in a right-sided downward direction would be the main mechanism to explain this phenomenon (Fig 4).

When a head tilt was applied, extension of the atlanto-occipital and cervical spine joints occurred first. After these joints had locked, the chest (sternal notch) began to rise. When the atlanto-occipital and cervical spine joints locked, no further angulation was possible between the laryngeal and oral cavity axes. As such, any further increase in flow rate could not be accomplished through increasing the HT/CL angle. Based on this constraint, we presumed that the benefits of the HT/CL maneuver to open the upper airway would be greatest at that point. Additionally, this suggestion may be applicable in the same way to obtunded or paralyzed patients who are likely to be angulated more than conscious participants because they also show chest (sternal notch) rise when angulation became bigger and bigger. In other words, it will be better for providers to applicate maximal HT/CL angulation as far as they can, till the patients’ chest (sternal notch) begin to rise.

**Fig 5:**
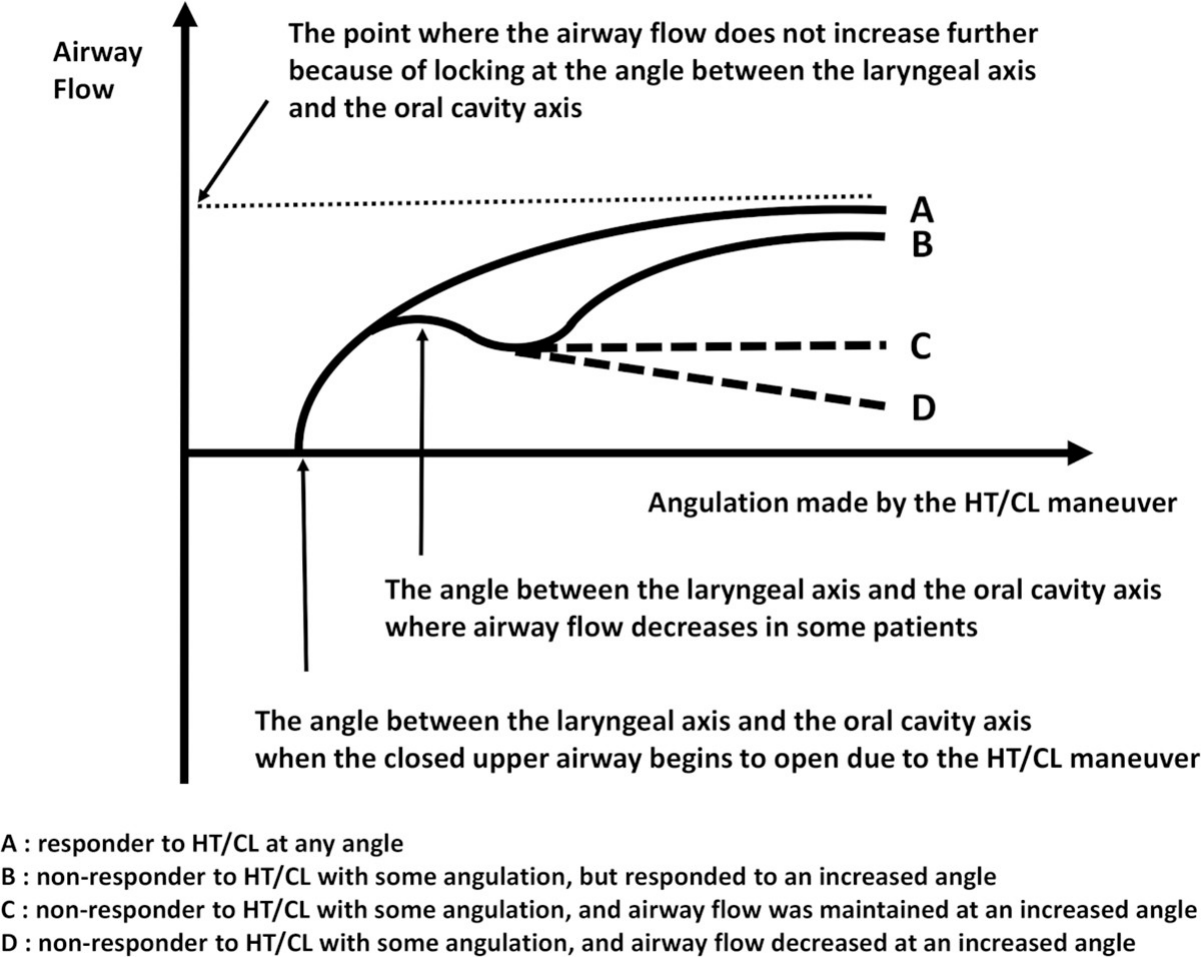
This graph shows the airway flow according to the HT/CL angulation. The HT/CL angulation was calculated as 90° minus the angle between the ear-eye line and the horizontal line. Abbreviation: HT/CL, head-tilt/chin-lift maneuver.

Another notable and unique finding in this study concerns responsiveness to the HT/CL maneuver. In our previous study, 16.6% of participants were non-responsive to the HT/CL maneuver. The present study also identified nearly the same percentage of non-responders among the total number of participants. However, many non-responders became responsive to the HT/CL maneuver when the HT/CL angle was increased. This was clinically significant as airway resistance could be increased through a slight HT/CL angulation in some patients; however, the mechanism through which this occured has yet to be identified. This result is shown in line B, Fig 5. It appears beneficial to perform the HT/CL maneuver to the greatest extent possible to minimize upper airway resistance, which means performing the maneuver to the point where locking the extended atlanto-occipital and cervical spine joints occurs and the chest (sternal notch) begins to rise. Some non-responders to HT/CL #1 showed a constant or decreased airway flow rate at HT/CL #2 (C and D at Fig 5); however, the significance of this finding is unclear.

### Limitations

Our study had some limitations. First, this study failed to determine variables to predict the responsiveness to the HT/CL maneuver. Basic variables in regard to general body morphology did not differ, nor did they differ in our previous study. LMD, MHD, and HTD, collected to reflect upper airway characteristics, did not differ between groups. We consider that characteristics relating to the extension of the atlanto-occipital and cervical spine joints would be promising discriminators between responders and non-responders to the HT/CL maneuver. Second, radiographic evaluations were not performed, which might possibly have revealed more precise axial correlations. Additionally, an endoscopy evaluation would have been helpful to evaluate the intraluminal conditions. Third, the sample size was too small to generalize the percentage change in the PEFRs. Moreover, the study participants were 20 years old; therefore, whether the percentage change in the PEFR would be similar among other population age groups remains uncertain. Nevertheless, we were able to derive a clinically important recommendation from this study, that is, to tilt the head and lift the chin until a patient’s chest (sternal notch) begins to rise.

## Conclusions

Our findings suggest that a greater HT/CL angle would be beneficial, as the PEFR increased gradually. Some participants did not respond to HT/CL #1, however, the PEFR increased or declining trend slowed down in many non-responders at post-HT/CL #2. The decreasing trend in the PEFR increase with the HT/CL angle implies the existence of an angle threshold beyond which there is no further benefit in airflow, indicating a minimum in airway resistance. A HT/CL maneuver may be appropriate until locking the atlanto-occipital and cervical spine joints in extension occurs and the chest (sternal notch) begins to rise.

## References

[1] Safar P, Escarraga LA, Chang F. Upper airway obstruction in the unconscious patient. J Appl Physiol. 1959;14:760–764. 10.1152/jappl.1959.14.5.760 14440737

[2] Zideman DA, De Buck ED, Singletary EM, Cassan P, Chalkias AF, Evans TR, et al European Resuscitation Council Guidelines for Resuscitation 2015 Section 9. First aid. Resuscitation. 2015;95:278–287. 10.1016/j.resuscitation.2015.07.031 26477417

[3] Perkins GD, Handley AJ, Koster RW, Castrén M, Smyth MA, Olasveengen T, et al European Resuscitation Council Guidelines for Resuscitation 2015: Section 2. Adult basic life support and automated external defibrillation. Resuscitation. 2015;95:81–99. 10.1016/j.resuscitation.2015.07.015 26477420

[4] Kleinman ME, Brennan EE, Goldberger ZD, Swor RA, Terry M, Bobrow BJ, et al Part 5: Adult Basic Life Support and Cardiopulmonary Resuscitation Quality: 2015 American Heart Association Guidelines Update for Cardiopulmonary Resuscitation and Emergency Cardiovascular Care. Circulation. 2015;132:S414–35. 10.1161/CIR.0000000000000259 26472993

[5] Jo S, Lee JB, Jeong T, Jin Y, Yoon J, Park B. Change in peak expiratory flow rate after the head-tilt/chin-lift maneuver among young, healthy, and conscious volunteers. Clin Exp Emerg Med. 2019;6: 36–42. 10.15441/ceem.18.006 30944290 PMC6453697

[6] Safar P. New data on resuscitation. IEEE Transactions on Power Apparatus and Systems. 1958;77:781–3

[7] Safar P, Escarraga LA, Chang F. Upper airway obstruction in the unconscious patient. J Appl Physiol. 1959;14:760–764. 10.1152/jappl.1959.14.5.760 14440737

[8] Guildner CW. Resuscitation: opening the airway. A comparative study of techniques for opening an airway obstructed by the tongue. JACEP.1976;5:588–590. 1018384 10.1016/s0361-1124(76)80217-1

[9] Greene DG, Elam JO, Dobkin AB, Studley CL. Cinefluorographic study of hyperextension of the neck and upper airway patency. JAMA. 1961;176:570–573. 10.1001/jama.1961.03040200006002 13708290

[10] Ruben HM, Elam JO, Ruben AM, Greene DG. Investigation of upper airway problems in resuscitation. 1. Studies of pharyngeal x-rays and performance by laymen. Anesthesiology. 1961;22:271–279. 10.1097/00000542-196103000-00017 13744316

[11] Elam JO, Greene DG, Schneider MA, Ruben HM, Gordon AS, Hustead RF, et al Head-tilt method of oral resuscitation. J Am Med Assoc. 1960;172:812–815. 10.1001/jama.1960.03020080042011 13819856

